# Foliar Nanoparticulate Sulphur and Amino Acids Modulate Wheat Yield Components and Seed Quality Across Contrasting Environments

**DOI:** 10.3390/plants15010066

**Published:** 2025-12-25

**Authors:** João Pedro Chacon Pereira, Letícia Elisiane Beluzzo, Gabriela da Silva Machineski, Claudemir Zucareli, Adônis Moreira, Halley Caixeta Oliveira, Inês Cristina de Batista Fonseca

**Affiliations:** 1 Postgraduate Program in Agronomy, Department of Agronomy, State University of Londrina (UEL), Londrina 86057-970, PR, Brazil; joaopedrochaconpereira@gmail.com (J.P.C.P.); leticiabeluzzo@gmail.com (L.E.B.); gs.machineski@uel.br (G.d.S.M.); claudemircca@uel.br (C.Z.); 2 Embrapa Soja, Carlos João Strass Highway, s/nº Orlando Amaral Access, Warta District, Londrina 86085-981, PR, Brazil; adonis.moreira@embrapa.br; 3 Department of Animal and Plant Biology, State University of Londrina (UEL), Londrina 86057-970, PR, Brazil

**Keywords:** *Triticum aestivum* L., biostimulants, soil fertility, seed quality, sustainable agriculture

## Abstract

Wheat productivity and seed quality are often constrained by nutrient imbalances and environmental stress, which can be mitigated through biostimulants and nanofertilisers. This study evaluated the effects of foliar applications of nanoparticulate sulphur (SNP) and hydrolysed amino acids (AAs) on wheat agronomic performance and seed quality under contrasting environmental conditions in Paraná, Brazil, during the 2022 and 2023 growing seasons. The experiment was conducted in four environments, using a randomised block design, with a 5 × 2 factorial scheme and four replications. Parameters included grain yield, yield components, and physiological and nutritional seed traits. SNP positively influenced the number of grains per ear and spikes per metre, with quadratic responses peaking at 1.048 kg ha^−1^ for SNP and 0.347 kg ha^−1^ for S, respectively. However, AAs showed no significant effects, likely due to favourable climatic conditions and high soil fertility. Regarding seed quality, a positive response in seed vigour was observed at 2 kg ha^−1^ SNP in one environment, while other parameters showed no consistent improvement. Principal component analysis indicated that environment and soil fertility were the main sources of variation in yield and seed quality. Overall, foliar SNP and AA applications did not markedly enhance wheat performance under non-stressful conditions.

## 1. Introduction

The introduction of wheat *(Triticum aestivum* L.) is one of the most widely cultivated cereals and a cornerstone of global food security, supplying a substantial share of dietary energy and protein [1,2]. Although advances in breeding and crop management have boosted production, yield gains are still constrained by climatic variability and heterogeneous soil fertility across tropical and subtropical regions. Environmental conditions, especially water availability and nutrient status, govern nutrient assimilation, photosynthesis, and the formation of yield components [1,3,4].

Among macronutrients known to be essential, sulphur (S) plays a pivotal role in plant metabolism. It is required for the synthesis of cysteine and methionine, for coenzymes and vitamins, and for maintaining cellular redox homeostasis via glutathione-dependent pathways. An adequate supply of S sustains the N:S ratio necessary for protein synthesis, chlorophyll formation, nitrogen use efficiency, and grain quality in wheat; conversely, an S deficiency leads to chlorosis, accumulation of non-protein nitrogen, and impaired technological quality [3,4,5,6,7]. The decline in atmospheric S deposition and the widespread use of S-free high-analysis fertilisers have increased the frequency of S deficiency in many regions. In this context, foliar fertilisation offers rapid, targeted nutrient delivery at critical phenophases (e.g., booting/anthesis), and can bypass soil constraints such as low S mineralisation or leaching; foliar S may enhance N assimilation, protein accumulation, and bread-making quality, particularly when root uptake is restricted [4,8,9]. In tropical and subtropical production systems, soils often show medium to high baseline S fertility and long fertilisation histories, meaning that additional yield responses to foliar S or biostimulants can be limited under these conditions [10,11].

Nanotechnology provides additional opportunities to improve nutrient use efficiency. Nanofertilizers exhibit a high specific surface area, enhanced reactivity, and in some cases, controlled-release behaviour. Nanoparticulate sulphur (SNP) has been proposed to improve cuticular penetration and residence on the leaf surface, enabling efficient physiological responses at lower application rates [12,13,14,15,16]. Experimental evidence indicates that SNP can promote photosynthetic performance, growth, and tolerance to salinity, drought, and heavy-metal stress, with potential benefits for biofortification and reduced environmental losses [13,14,15,16]. Nanoparticulate S typically exhibits enhanced leaf retention and higher surface reactivity, which can promote more effective physiological responses at lower application rates compared with bulk S formulations [17,18]. However, most studies have evaluated SNP in isolation; integrative approaches combining SNP with biostimulants are largely unexplored, despite the potential for synergistic enhancement of nutrient assimilation and stress resilience [19,20]. However, replicated multi-environment field studies remain scarce, particularly those linking agronomic responses to seed physiological and nutritional outcomes under realistic management.

In parallel, amino acid (AA)–based biostimulants and protein hydrolysates (PHs) have gained prominence for enhancing plant performance via multiple mechanisms. Beyond structural roles, AAs act as signalling and osmoprotective molecules, modulating photosynthesis, nutrient uptake, C–N metabolism, and antioxidant defences [21,22,23,24,25,26,27]. Among the predominant AAs in hydrolysates, glycine contributes to photorespiratory fluxes and redox regulation [28,29]; glutamic acid functions as a central hub in N assimilation and signalling pathways [30]; and proline acts as an osmoprotectant, stabilising membranes and scavenging reactive oxygen species under abiotic stress [31,32]. Documented benefits include improved growth, micronutrient uptake (e.g., Zn, Fe, Mn), and resilience to abiotic stresses; however, the magnitude and consistency of responses are highly environment-dependent and sensitive to timing, dose, and intrinsic soil fertility [21,22,26,27]. Given these complementary modes of action, combining SNP with AAs may yield synergistic effects; yet, to date, integrative field evidence, particularly connecting yield components with seed physiological and nutritional quality, remains limited.

Although the individual effects of S fertilisation, nanofertilisers, and amino acid–based biostimulants are well documented, studies assessing their combined action remain limited. Most available evidence focuses on isolated applications, offering little insight into potential synergistic outcomes or their influence on seed physiological and nutritional traits. This scarcity is particularly evident in tropical and subtropical environments, where soil and climate variability can strongly shape treatment responses [12,13,14,15,16,22,23,24,25,26,27]. Therefore, evaluating the interaction between SNP and AAs under realistic field conditions is essential to advance current understanding.

Because nutrient × environment × management interactions are complex, the current study evaluated the combined effects of foliar-applied SNP and AAs on (i) yield components and grain yield, (ii) seed physiological quality, and (iii) seed nutrient composition of wheat across four contrasting environments. We hypothesised that (a) SNP would enhance yield components and seed vigour, particularly under lower S availability, (b) AAs would amplify responses under less favourable environments, and (c) treatment responses would be strongly context dependent.

## 2. Results and Discussion

### 2.1. Response Variables and Sampling

The combined analysis of variance across four environments (2022–2023 growing seasons) detected no significant effects of foliar-applied sulphur nanoparticles (SNP), hydrolysed amino acids (AAs), or their interaction (SNP × AA) on grain yield (YLD) (Table 1). Soil S levels ranged between 13 and 31.13 mg dm^−3^, considered adequate to high for wheat, which reduces the probability of a response to foliar supplementation [23,33]. These findings are consistent with reports indicating that gains from foliar S occur mainly under soil nutrient limitation [34,35] or when tissue N:S ratios are unbalanced [36]. In situations where this ratio is already in equilibrium, further yield increases are unlikely, and the high nutrient availability in the root zone may even suppress foliar uptake, especially in crops with waxy leaves such as wheat [23]. Physiologically, the wheat response to S is tightly linked to its role in S amino acid synthesis and redox regulation; however, when basal S and N levels are sufficient to sustain protein synthesis and grain quality, the crop tends not to show additional yield gains [5,6,7].

A potential factor contributing to the lack of response observed in this study is the topdressing strategy, which involved the application of 200–300 kg ha^−1^ of ammonium sulphate. This practice supplies a substantial amount of readily available sulphate-S, ensuring that rhizosphere S availability remains fully adequate throughout vegetative growth. Under such conditions, additional foliar S applied at anthesis is unlikely to elicit further physiological or yield benefits. This interpretation is supported by [37], who highlighted the rapid release and high plant availability of sulphate-S from ammonium sulphate, and by [38], who demonstrated that ample soil S availability markedly decreases the probability of crop responses to supplementary S sources.

**Figure 1:**
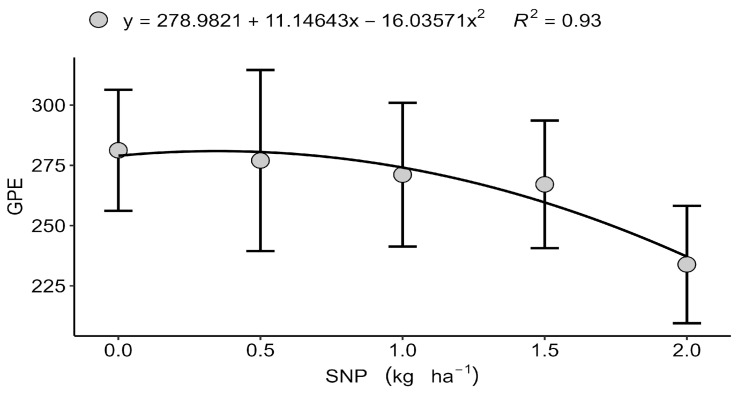
Isolated effect of the nanoparticulate sulfur (S) dose on the number of grains per ear (GPE), Londrina–PR, 2022. Data are presented as mean ± standard deviation (*n* = 4).

**Figure 2:**
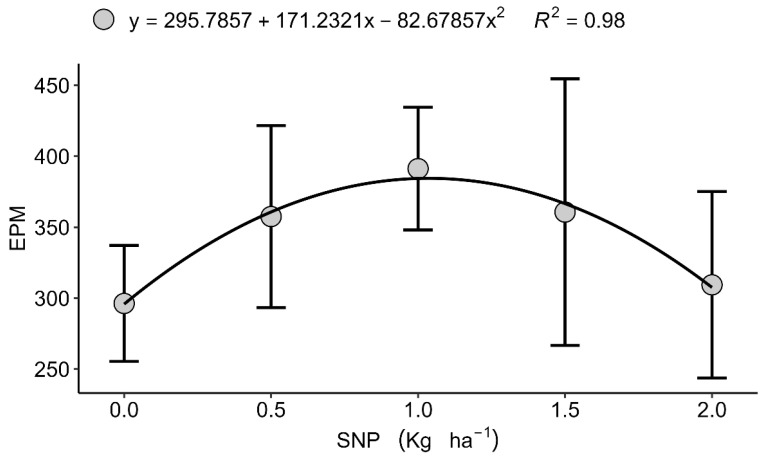
Isolated effect of the nanoparticulate sulphur (S) dose on the number of ears per square metre (EPM), Faxinal, PR, 2023. Data are presented as mean ± standard deviation (*n* = 4).

Although grain yield itself did not respond to treatments, significant effects were observed in specific yield components. In Londrina (2022), the number of grains per ear (GPE)followed a quadratic trend, reaching a maximum at 0.347 kg ha^−1^ of S (Figure 1) before declining at higher doses, which suggests a physiological limitation or nutritional antagonism under excessive S input. Excess S, particularly in nanoparticulate form, may disrupt the cellular redox balance and interfere with the assimilation of other nutrients, such as N and Se, potentially affecting grain filling [16,39]. Similarly, in Faxinal (2023), the number of of ears per square metre (EPM) showed a quadratic response, with an optimum value of 1.048 kg ha^−1^ SNP (Figure 2). However, the increase in spikes was not accompanied by higher grain yield, indicating compensatory adjustments among yield components including spike number, grain number, and thousand-grain weight, which highlights the central role of S in grain filling rather than spike initiation [13,40]. These results are consistent with previous studies showing that biostimulants and nutrient inputs often have marginal effects under non-stress conditions, with benefits being more pronounced under nutrient or environmental limitations [41,42].

The absence of significant effects of AAs on agronomic variables and seed physiological quality is also compatible with the favourable growing conditions during the experimental years (18–23 °C, RH > 70%, and regular rainfall), under which the metabolic signalling effects of amino acids tend to be subtle. Their primary role as signalling and osmoprotective molecules is expressed more strongly under abiotic stress, where they can enhance photosynthesis, antioxidant defence, and nutrient assimilation [21,22,41,43,44]. Under optimal conditions, plants channel resources to vegetative growth and reproductive development, reducing the expression of AA-triggered stress-adaptation pathways [26,27].

Mechanistically, the limited response to foliar-applied S may be partly explained by the need for S^0^ oxidation to SO_4_^2−^ prior to assimilation, a process more efficient in the soil environment than in the phyllosphere, which is microbially oligotrophic [13,45]. Moreover, the physicochemical properties of the formulation, including particle size, surfactants, and encapsulation, are critical for cuticular penetration, leaf surface retention, and symplastic transport [46,47]. Even when yield components are modulated, their effect on final yield depends on the synchrony between nutrient availability and the crop’s phenological stage, as well as potential synergies with nitrogen supply [7,48].

**Figure 3:**
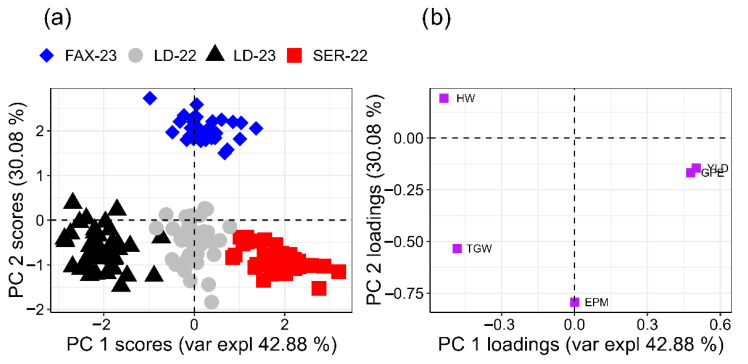
Principal component analysis (PCA) of agronomic variables: number of ears per square metre (EPM), number of grains per ear (GPE), weight per thousand grains (g) (WTG), weight of hectolitre (kg hL^−1^) (HW), and grain yield (kg ha^−1^) (YLD). across four wheat-growing environments. (**a**) Score plot showing the distribution of experimental environments: LD-22 (Londrina 2022), LD-23 (Londrina 2023), SER-22 (Sertaneja 2022), and FAX-23 (Faxinal 2023). (**b**) Loading plot illustrating the contribution of variables to the first two principal components (PC1 and PC2).

Multivariate analysis supported these observations: the agronomic PCA explained 72.45% of the total variance (PC1 = 42.37%; PC2 = 30.08%) and revealed that environment and growing season exerted a far greater influence on trait expression than the applied treatments (Figure 3). PC1 was predominantly associated with yield-related variables such as spike number per metre and grain yield, suggesting that this axis reflects differences in overall resource availability, soil fertility, and climatic favourability among sites. In contrast, PC2 was mainly defined by grains per spike and plant height, representing variation in reproductive development and canopy architecture. This orthogonal separation between productivity-driven (PC1) and morphological/reproductive components (PC2) aligns with previous reports on the genotype × environment interaction in wheat [49,50,51].

Together, these patterns reinforce that in S-sufficient soils and under non-stressful growing conditions, foliar SNP and AAs have limited capacity to modify the multivariate structure of agronomic performance. The score plot showed no visible clustering by treatment, whereas the environments grouped distinctly, indicating that edaphoclimatic differences, particularly rainfall distribution, temperature regimes, altitude, and intrinsic soil fertility, dominated the response [11,12].

From a practical standpoint, the use of foliar S (including nanoparticulate forms) and amino acids should be strategically targeted. Recommendations should be based on prior soil and tissue diagnostics, with attention to N:S ratios and clear agronomic objectives, such as grain quality improvement or specific yield components. The selection of appropriate formulations, especially regarding nanoparticle dispersion and surfactant systems and the timing of application are crucial to maximise absorption and minimise antagonistic interactions. Furthermore, multi-environment validation is essential, prioritising nutrient-limited or stress-prone scenarios, where the probability of a positive agronomic response is substantially higher [40,41,46,47]. When appropriate, integrative management strategies combining S and N supply should be considered to optimize protein synthesis and grain filling [7,48].

Across four contrasting environments with adequate-to-high soil sulphur [15,16,17,18,19,20,21,22,23,24,25,26,27,28,29,30,31,32,33,34,35] (13 mg dm^−3^), foliar nanoparticulate sulphur (SNP) and amino acids (AAs) did not upscale to grain-yield gains, underscoring the primacy of baseline fertility and edaphoclimatic context in conditioning treatment efficacy. This aligns with agronomic guidance that foliar S responses are most probable under edaphic limitation or tissue N:S imbalance [23,33,34,35,36]. In such S-sufficient settings, the physiological roles of S in cysteine/methionine synthesis and redox buffering remain fulfilled, and further inputs rarely translate into additional yield [5,6,7]. Nevertheless, the detection of component-level responses within narrow dose windows, peak grains per spike in Londrina at a moderate SNP rate and a quadratic rise in spike number per metre in Faxinal, indicates that SNP can modulate developmental trajectories when the signal is well timed and dosed, even if whole-plant compensation masks final yield effects. The decline in grain set beyond the optimum is consistent with redox perturbation and competitive interactions in shared transport/metabolic pathways (e.g., with N and Se) at higher S loads [16,39], while the spike-number response that failed to convert into yield reflects classic compensation among components (spike number, grains per spike, thousand-grain weight) and reinforces that S is more consequential during grain filling than during spike initiation [13,40].

In the tropical and subtropical production systems where our study was conducted, soils generally exhibit medium to high baseline S fertility [11,12]. This condition was further strengthened by the application of 200–300 kg ha^−1^ of ammonium sulphate in topdressing, which supplied a substantial and rapidly available source of sulphate-S. Collectively, these factors ensured adequate S availability throughout vegetative growth, substantially reducing the likelihood that additional foliar S applied at anthesis would produce further physiological or yield effects. This interpretation aligns with evidence showing that sulphate-S from ammonium sulphate is rapidly plant-available [37] and that, when the soil already meets crop S demand, responses to supplementary S inputs become highly unlikely [38].

### 2.2. Seed Physiological Quality and Nutrient Analyses

**Figure 4:**
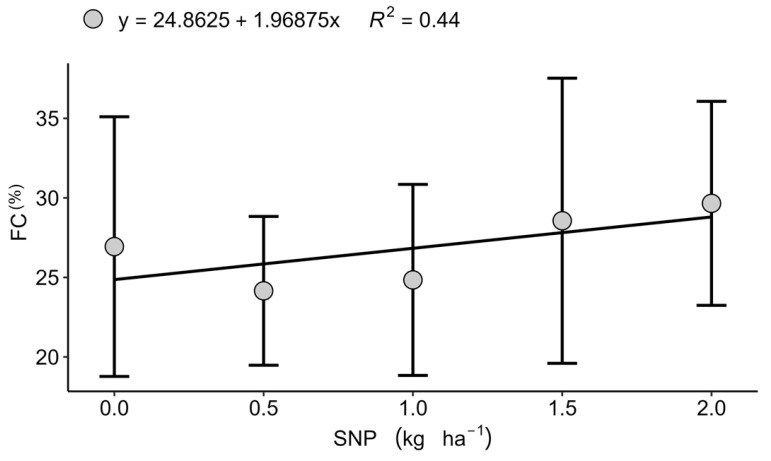
Effect of nanoparticulate sulphur (SNP) doses on first count (FC) of wheat seeds (%) in Sertaneja, PR, during the 2022 growing season. Values represent mean ± standard deviation (*n* = 4).

Foliar application of sulphur nanoparticles (SNP) and amino acids (AAs) did not significantly affect the majority of physiological seed quality parameters (Table 2). An exception was observed in Sertaneja (2022), where seed vigour, assessed by the first count test, exhibited a quadratic response, with an optimum value at 2 kg ha^−1^ SNP (Figure 4), indicating a beneficial effect under moderate fertility. Mechanistically, S is necessary for the biosynthesis of cysteine and methionine, amino acids essential for structural proteins and antioxidant/defence enzymes during germination, thereby sustaining early seedling metabolism when supplied at moderate rates [52,53,54].

No significant AA effects were detected for physiological traits across environments. which is consistent with the low incidence of abiotic stress during the growing seasons (18–23 °C, RH > 70%, and regular rainfall). Under non-limiting conditions, AA-driven signalling and osmoprotection are less expressed, explaining the muted responses observed here [21,41,42,54,55,56]. This reinforces the context dependence of biostimulants, with larger effects typically reported under suboptimal environments. (Table 3).

**Figure 5:**
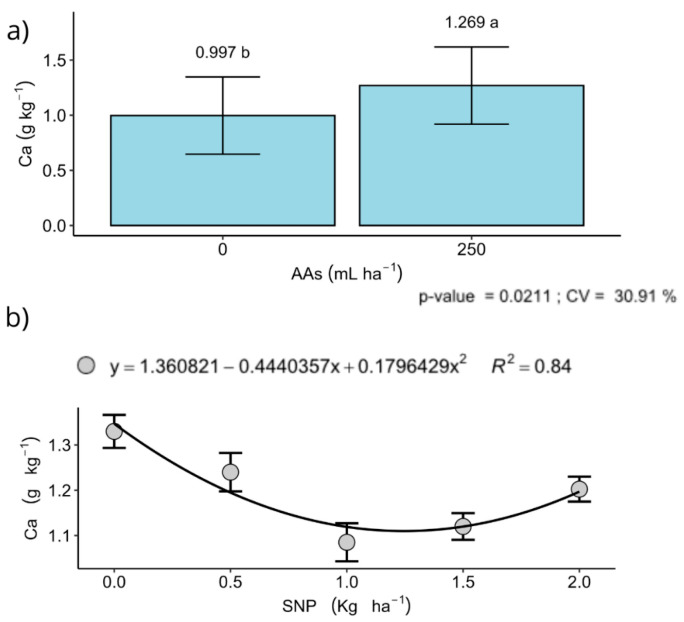
Isolated effects of: (**a**) amino acid and of (**b**) nanoparticulate sulphur (S) doses on calcium (Ca) content in wheat grains (g kg^−1^). Data obtained in the municipality of Londrina, PR, during the 2022 season. Data are presented as mean ± standard deviation (*n* = 4). Different lowercase letters in panel (**a**) indicate significant differences among treatments according to the Tukey test (*p* ≤ 0.05). Panel (**b**) represents a polynomial regression analysis.

Regarding mineral composition, seed calcium (Ca) was significantly influenced by the main effect of SNP and by its interaction with AAs in Londrina and Sertaneja (2024). In Londrina, AAs increased seed Ca (Figure 5a), supporting the view that biostimulants may enhance nutrient acquisition and internal redistribution [57]. In contrast, in Sertaneja, the highest Ca concentration occurred in the untreated control (0 kg ha^−1^ SNP) (Figure 5b), suggesting nutrient dilution or antagonism at higher SNP rates, underscoring the need for balanced S–Ca management. Other seed nutrients (P, K, Mg, Zn) showed no significant responses to SNP, likely reflecting the high baseline fertility in Londrina and Faxinal. In Sertaneja, the apparent reduction in Ca use efficiency at higher SNP rates points to a potential nutrient imbalance that merits further investigation.

A reduction in seed Ca concentration was observed in Sertaneja when comparing the control with higher SNP doses (Figure 5b), indicating a consistent decline in Ca content under increased S supply. This pattern is more plausibly explained by ionic antagonism between S and divalent cations such as Ca than by simple nutritional dilution. Excess S availability has been associated with reduced Ca uptake, translocation or storage in several crops [58], and fertilisation studies report that high S inputs or S-containing fertilisers can depress Ca accumulation even when soil Ca availability is adequate [5]. Because Ca plays a key role in cell-wall stability and grain-filling processes, such antagonism may have restricted Ca delivery to developing grains, preventing favourable effects of SNP on yield components (e.g., grains per ear) from translating into higher final grain yield.

A similar context dependence emerged for AAs. Under favourable temperature, humidity, and rainfall, the signalling and osmoprotective functions of AA-based biostimulants remained largely latent, in line with evidence that their agronomic benefits are clearest under abiotic constraints such as drought, salinity, or thermal stress [21,22,41]. This helps reconcile the mixed literature on AAs: in optimal conditions plants prioritise growth and reproduction, attenuating the need for stress-priming pathways that AAs typically activate [20,26]. Where a clear physiological signal did surface, increased seed vigour at 2 kg ha^−1^ SNP in Sertaneja, the response is coherent with S-driven enhancement of sulphur-containing amino acids and antioxidant machinery during germination [52,53,54]. Conversely, the decline in seed Ca at higher SNP rates at the same site suggests either dilution with larger seed biomass or ionic antagonism, highlighting the need to balance S with divalent cations during reproductive stages.

**Figure 6:**
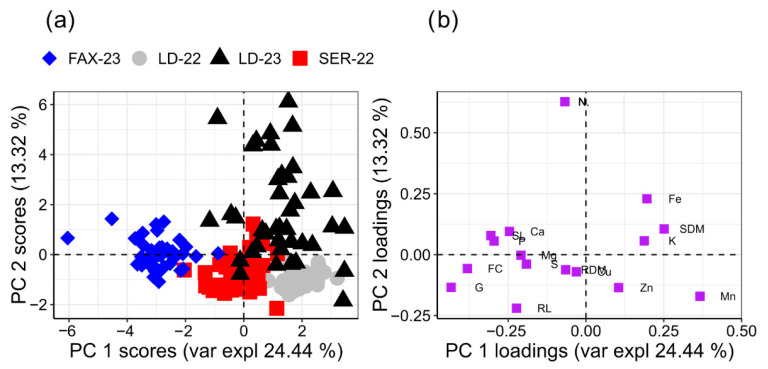
Principal component analysis (PCA) of the relationships between seed chemical attributes, nutrient contents (Nitrogen–N (g kg^−1^), Phosphorus—P (g kg^−1^), Potassium—K (g kg^−1^), Magnesium—Mg (g kg^−1^), Calcium—Ca (g kg^−1^), Sulphur—S (g kg^−1^), Copper—Cu (mg kg^−1^), Iron—Fe (mg kg^−1^), Manganese—Mn (mg kg^−1^), and Zinc—Zn (mg kg^−1^)), and physiological seed quality traits (germination—G (%), first germination count—FC (%), emergence speed index—ESI, shoot length—SL (cm), root length—RL (cm), shoot dry mass—SDM (g plant^−1^), and root dry mass—RDM (g plant^−1^)) in wheat across four environments. (**a**) Score plot showing the distribution of experimental environments: LD-22 (Londrina 2022), LD-23 (Londrina 2023), SER-22 (Sertaneja 2022), and FAX-23 (Faxinal 2023). (**b**) Loading plot illustrating the contribution of variables to the first two principal components (PC1 and PC2).

As part of the second PCA, conducted independently from the agronomic PCA and focused specifically on physiological and mineral seed traits, environmental conditions again emerged as the dominant drivers of multivariate variation. No clustering associated with SNP or AA doses was observed (Figure 6). PC1 (24.44%) captured differences primarily related to micronutrients (Fe, Mn, Zn), indicating that soil redox behaviour, moisture regime, and texture were the key factors distinguishing environments. PC2 (13.32%) was defined by Ca, Mg and early seedling traits (SL, RL, ESI), reflecting the influence of base-cation availability and local edaphoclimatic conditions on early seedling development.

Seed physiological variables such as germination (G), first count (FC), and root dry mass (RDM) remained close to the origin, demonstrating biological stability under the favourable temperature and moisture conditions of the study years. Their limited contribution to multivariate separation is consistent with the ANOVA results, which showed minimal treatment effects and aligns with evidence that AA- and S-mediated responses are more pronounced under abiotic stress [21,22,41,55].

Together, the PCA indicates that both nutrient accumulation and seed physiological quality were shaped primarily by environmental gradients rather than by foliar SNP or AA inputs. Micronutrient dynamics governed PC1, while PC2 reflected cation balance and early seedling performance, and neither axis was substantially altered by foliar treatments. These results reinforce that SNP and AAs are more likely to produce measurable physiological gains under nutrient-limited or stress-prone conditions [59,60].

From a practical perspective, the findings highlight the importance of targeting these inputs to environments with diagnosed deficiencies or stress exposure, and ensuring appropriate formulation characteristics, application timing, and N:S balance to avoid antagonisms and maximize treatment efficiency [5,48,61].

Mechanistic constraints help explain why foliar S sometimes fails under field conditions. Elemental S must be oxidised to sulphate (SO_4_^2−^) prior to assimilation, a transformation that proceeds more effectively in soils than on the leaf surface because the phyllosphere is microbially oligotrophic; consequently, formulation and delivery, surfactants, encapsulation, and particle-size control and dispersion, become decisive determinants of cuticular passage, residence time and within-leaf transport [13,45,46,47]. These constraints, coupled with adequate soil supply, make it unsurprising that only specific components respond and only within tight dose–timing windows. Multivariate patterns are consistent with this interpretation: environment and season, rather than treatments, explained most variance, with spike number and yield tracking the primary axis and grains per spike and plant height loading the secondary axis, a structure frequently reported for wheat under strong environment × management control [49,50,51,60].

Three implications follow for practical applications. First, soil S diagnostics and tissue N:S ratios are essential to avoid low-probability interventions in S-sufficient fields [23,33,36]. Second, synchronising S applications with late vegetative or early reproductive stages, especially when grain filling or protein quality is the target, may provide more consistent benefits [7]. Third, formulation–phenology matching is critical: using dispersion systems (surfactants/encapsulation) and particle sizes that enhance cuticular penetration, applied under favourable microclimatic conditions, improves uptake and mitigates antagonisms [13,46,47]. These principles are particularly relevant in tropical and subtropical systems, where soils often exhibit medium to high baseline S fertility due to long fertilisation histories. In our study, this sufficiency was further reinforced by the topdressing of 200–300 kg ha^−1^ of ammonium sulphate, a rapidly plant-available source of sulphate-S, ensuring full rhizosphere adequacy during vegetative growth. Under such conditions, additional foliar S at anthesis is unlikely to produce physiological or yield gains, consistent with evidence that ample soil S markedly reduces crop responsiveness to supplementary S inputs [11,37,38]. Overall, our results indicate that SNP and AAs should be deployed selectively in nutrient-limited or stress-prone environments, where the probability of agronomic return is higher.

## 3. Materials and Methods

### 3.1. The Materials Field Trials and Experimental Design

Field trials were conducted across four cultivation environments during the winter seasons of 2022 and 2023 in Paraná State, Brazil: Londrina (2022 and 2023), Sertaneja (2022), and Faxinal (2023). In Londrina, the soil is classified as an Eutroferric Red Nitosol (LVe), medium texture (≈650 ± 50 g kg^−1^ clay) on gently undulating to flat terrain. In Sertaneja and Faxinal, the soils are Eutroferric Red Latosols with approximately 65% clay, 25% silt, and 10% sand. Site elevations are ~560 m (Londrina), ~520 m (Sertaneja), and ~820 m (Faxinal). All locations are characterised by a Köppen–Geiger Cfa climate (humid subtropical, hot summers) [62].

Prior to beginning the trial, composite soil samples were collected and analysed by certified laboratories; the summary baseline soil chemistry for each environment is presented in Table 4.

At every site, experiments followed a randomised complete block design (RCBD) arranged as a 5 × 2 factorial, comprising five doses of nanoparticulate sulphur (SNP) crossed with the presence/absence of amino acids (AAs), with four replicates per treatment. Each plot consisted of 14 rows, 7 m in length, spaced 0.17 m apart (plot area ≈ 16.66 m^2^). The six central rows constituted the net harvested area (useful area ≈ 7.14 m^2^).

**Figure 7:**
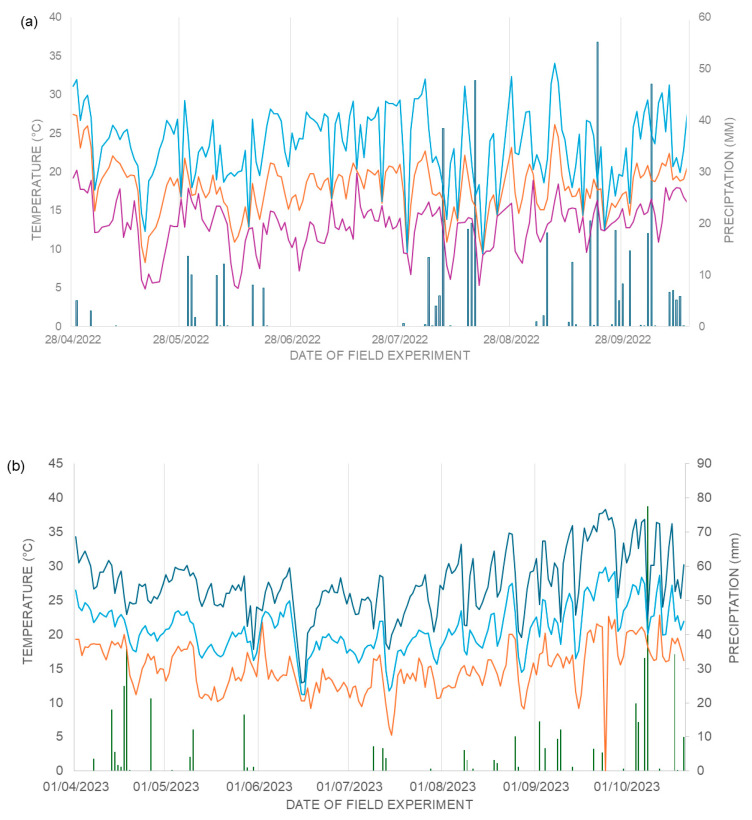
Graph of climatic data: (**a**) Municipality of Londrina and Sertaneja, during the experimental period of the second harvest of the year 2022, daily precipitation in mm (dark blue bar), air temperatures in °C: maximum (light blue line), minimum (purple line) and average (orange line), and (**b**) Municipality of Londrina and Faxinal, during the experimental period of the second harvest of the year 2023, daily precipitation in mm (green bar), air temperatures in °C: maximum (dark blue line), minimum (orange line) and average (light blue line). Londrina-PR, 2023.

Daily weather data (rainfall; maximum, minimum, and mean air temperature) were obtained from IDR-PR meteorological stations for Londrina and Sertaneja. For Sertaneja (2022) and Faxinal (2023), the Londrina station was used as the nearest operational reference. Summary climate profiles for each site–year are shown in Figure 7.

### 3.2. Treatments and Foliar Applications

Nanoparticulate elemental sulphur (SNP) was applied at rates of 0, 0.5, 1.0, 1.5, and 2 kg ha^−1^ as a concentrated aqueous suspension (200 mL L^−1^) containing 1% total N (13.6 g L^−1^), 50% elemental S (690 g L^−1^), and a density of 1.38 g cm^−3^. The product was manufactured via top-down wet milling of 200-mesh elemental sulphur using zirconia–yttria beads, yielding ~80% of particles between 100 nm and 1 µm. The suspension was stabilised with dispersants and wetting agents, maintaining homogeneity for up to 24 months.

The SNP rates used in this study were defined according to the commercial recommendation of the Nano-S^®^ product (Nutritem Industria e Comercio Ltda., Londrina, PR, Brazil). For cereals, the manufacturer indicates foliar applications of approximately 0.5 L ha^−1^, which corresponds to ~0.35 kg of elemental S per hectare (50% S; density 1.38 g cm^−3^). Therefore, the selected rates (0–2 kg ha^−1^) encompass the recommended dose as well as sub- and supra-recommended levels, enabling a comprehensive dose–response assessment while maintaining agronomic relevance and consistency with practical field use.

The second experimental factor consisted of foliar application of amino acids (AAs), provided as a hydrolysate derived from animal collagen in liquid formulation, applied at a commercial rate of 250 L ha^−1^. The amino acid profile (e.g., glycine 13.5%, glutamic acid 5.6%, proline 8.3%) is provided in Supplementary Table S1.

The hydrolysate formulation was strategically designed to supply key metabolic precursors and biostimulant agents. The high concentration of glycine (13.5%) is beneficial, acting as a critical precursor for chlorophyll synthesis and enhancing micronutrient uptake efficiency through its documented chelating properties. Glutamic acid (5.6%) is vital in metabolism, serving as the primary entry point for nitrogen into amino acid synthesis while also supporting stomatal regulation via its role as a cytoplasmic osmotic agent. Furthermore, proline (8.3%) functions as a well-documented osmoprotectant, accumulating under abiotic stress to stabilize cellular membranes, enhance antioxidant defense, and maintain overall cellular integrity. This specific amino acid profile justifies the use of the hydrolysate to support growth and increase plant resilience.

Foliar applications were performed at pre-flowering/anthesis, at 45–50 growth stage [64] using a pressurised backpack sprayer equipped with flat-fan nozzles (XR 110.02, TeeJet^®^ Technologies, Cotia, SP, Brazil), delivering 200 L ha^−1^ of spray solution. Application procedures followed field best practices: neutral-quality water from the local supply, pre-mixing of the concentrate with manual agitation, addition of 0.5 L of surfactant adjuvant (Nutritem Industria e Comercio Ltda., Londrina, PR, Brazil), continuous agitation during spraying, and production of fine droplets for optimal leaf coverage.

### 3.3. Cultivars, Sowing Windows and Crop Management

The wheat cultivars used in each environment were selected based on local adaptation and disease resistance: TBIO Ponteiro in Londrina (2022 and 2023), RBO Combatente in Sertaneja (2022), and JVC Cerne in Faxinal (2023). Target plant populations were adjusted according to the cultivar, with 300–330 plants m^−2^ for TBIO Ponteiro and 240–260 plants m^−2^ for RBO Combatente and JVC Cerne, following regional recommendations.

In Londrina during 2022, sowing was carried out on 27 April following pre-sowing weed control with glyphosate (2 L ha^−1^) combined with 2,4-D (2 L ha^−1^) on 18 April. Post-emergence management included clodinafop-propargyl (2 L ha^−1^) applied on 6 June and 2,4-D at 2 L ha^−1^ on 15 June. Basal fertilisation consisted of 250 kg ha^−1^ of 10-15-15, and topdressing was performed with 200 kg ha^−1^ ammonium sulphate on 9 June. The harvest took place on 10 September 2022.

In Sertaneja during 2022, sowing was performed on 20 May after pre-sowing weed control with glyphosate (2 L ha^−1^) and 2,4-D (2 L ha^−1^) applied on 5 May, along with mowing of cotton residues. Post-emergence glyphosate (2 L ha^−1^) was applied on 6 June. Basal fertilisation was 600 kg ha^−1^ of 13-14-8, followed by topdressing with 300 kg ha^−1^ ammonium sulphate on 9 July. The harvest took place on 16 October 2022.

In Londrina during 2023, sowing occurred on 27 April, with pre-sowing weed control using glyphosate (2 L ha^−1^) and 2,4-D (2 L ha^−1^) on 6 April. Post-emergence applications of clodinafop-propargyl (Topic^®^) at 2 L ha^−1^ and 2,4-D (2 L ha^−1^) were carried out on 2 June and 13 June, respectively. Fertilisation management mirrored the previous year with 250 kg ha^−1^ of 10-15-15 as basal fertiliser and 200 kg ha^−1^ ammonium sulphate as topdressing on 12 June. The harvest took place on 10 September 2023.

In Faxinal during 2023, sowing was carried out on 25 May following pre-sowing weed control with glyphosate (2 L ha^−1^) on 5 May. Basal fertilisation consisted of 600 kg ha^−1^ of 10-15-15, and topdressing was performed with 300 kg ha^−1^ ammonium sulphate. Disease management included fungicide application with picoxystrobin + cyproconazole (0.6 L ha^−1^) on 22 August 2023. The harvest took place on 20 October 2023.

Overall, crop management practices at all sites adhered to the recommendations of the Brazilian Wheat Research Commission [65] for Paraná State, including fertilisation based on soil test results and standard integrated weed and pest management protocols

### 3.4. Response Variables and Sampling

At physiological maturity, plants from the central rows of each plot (useful area) were harvested manually and threshed using a stationary thresher. The number of ears per square metre (EPM) was determined by counting ears in four linear meters per plot. The grains per ear (GPE) was obtained by counting grains from ten randomly sampled ears per plot. Grain yield (YLD) was measured from the threshed grains of the useful area, weighed, and corrected to 13% moisture content, with results expressed in kg ha^−1^. Weight per thousand grains (g) (WTG) was determined by weighing two subsamples of 100 grains per plot and multiplying by ten, also corrected to 13% moisture. Hectolitre weight (HW) was measured using a Dalle Molle^®^ hectolitre scale (MOD Type 40, ¼ L cup, DM Equipamentos, Vinhedo, RS, Brazil) and converted to kg hL^−1^.

### 3.5. Seed Physiological Quality

Seed quality assessments were carried out after 60 days of storage under controlled conditions (10 °C, 50% relative humidity) at the Seed Technology Laboratory of the Universidade Estadual de Londrina (UEL). Germination tests followed the Rules for Seed Testing [66], using Germitest^®^ paper (Germilab, Passo Fundo, RS, Brazil) moistened with distilled water equivalent to 2.5 times the dry paper weight. Four replicates of 50 seeds per treatment were used. Normal seedlings were counted on the fourth and eighth days to determine first count (%) (FC) and final germination (%) (G).

Seedling vigour was further evaluated by measuring root length (cm) (RL) and shoot length (cm) (SL) of 10 normal seedlings per replicate with a millimetre ruler. Shoot dry mass (g) (SDM) and root dry mass (g) (RDM) was determined by oven-drying seedlings at 65 °C for 48 h and weighing them on an analytical scale.

For the emergence speed index (ESI), seeds were sown at a depth of 2 cm in trays filled with sterilised sand and maintained under controlled environmental conditions. Daily counts of emerged seedlings were performed for 15 days, and the ESI was calculated according to Maguire’s formula:

ESI = NPEd/En + NPEd/En + ∙∙∙ + NPEd/En

### 3.6. Seed Nutrient Analyses

Chemical analyses were conducted to determine the total concentrations of macronutrients (N, P, K, Ca, Mg, and S) and micronutrients (Cu, Fe, Mn, and Zn) in wheat seeds, following the methodologies described by [67]. For crude protein determination, total N was quantified by the Kjeldahl method, and protein content was calculated using a conversion factor of 5.7. For mineral analysis, grain samples (~30 g per plot) were surface-sanitised with sodium hypochlorite solution (10% NaOCl, 5 min), rinsed, oven-dried at 60 °C for 72 h, and finely ground. Subsamples were digested using a nitroperchloric acid mixture (HNO_3_:HClO_4_ = 3:1) and analysed by atomic absorption spectroscopy (AAS) for macro- and micronutrient quantification. Reference sufficiency ranges and interpretation criteria followed the official state fertilisation guidelines and literature standards for wheat seed composition.

### 3.7. Statistical Analyses

Data were checked for normality (Shapiro–Wilk) and homogeneity (Hartley). ANOVA (F-test) and dose-response regressions (linear/polynomial) were fitted at α = 0.05. Two separate principal component analyses (PCAs) were conducted: one including agronomic traits (yield and yield components) and another including physiological and mineral seed traits. As each PCA used a distinct variable set, the variance explained by PC1 and PC2 differed between analyses. All analyses were performed in R [68].

## 4. Conclusions

Foliar applications of nanoparticulate sulphur (SNP) and amino acids, whether applied individually or in combination, did not significantly influence wheat grain yield or most seed quality attributes under the high-fertility and non-stress conditions of the evaluated environments. Environmental and soil factors were the primary determinants of physiological and nutritional seed traits, and a detectable improvement in seed vigour occurred only at the highest S dose (2 kg ha^−1^) in a single location, reinforcing the strong context dependence of these inputs.

From a practical standpoint, the agronomic effectiveness of foliar SNP and amino acids appears limited in tropical soils with adequate S supply and favourable growing conditions. Their use should therefore be targeted to scenarios of diagnosed S deficiency or abiotic stress, where physiological responsiveness is more likely. These findings refine the positioning of S-based foliar biostimulants, highlighting that their application should be selective and guided by environmental diagnostics and crop nutritional status.

## Figures and Tables

**Table 1:** Summary of the analysis of variance for the characteristics of number of ears per square metre (EPM), number of grains per ear (GPE), weight per thousand grains (g) (WTG), weight of hectolitre (kg hl^−1^) (HW), and grain yield (kg ha^−1^) (YLD). Data obtained in the municipality of Londrina-PR in the 2022 harvest.

Causes of Variation	D.F.	*p*-Value				
		EPM	GPE	WTG (g)	HW (kg hl^−1^)	YLD (kg ha^−1^)
Londrina-PR in the 2022 Growing Season						
Amino acid (A)	1	0.1740	0.2439	0.3982	0.7238	0.9339
Sulphur Doses (D)	4	0.5954	0.0222 *	0.4111	0.9887	0.289
A × D	4	0.9124	0.7176	0.7738	0.6791	0.394
C.V. (5%)		15.98	10.83	29.19	3.95	15.15
Mean		368.5	266.07	20.9	71.7	1697.70
**Sertaneja-PR in the 2022 Growing Season**						
Amino acid (A)	1	0.9817	0.2734	0.4736	0.4736	0.445
Sulphur Doses (D)	4	0.4955	0.2405	0.4051	0.4051	0.2737
A × D	4	0.6028	0.5283	0.3377	0.3377	0.2945
C.V. (5%)		1.76	15.33	30.64	30.64	29.41
Mean		410.12	326.6	29.4	80.3	2418.74
**Londrina-PR in the 2023 Growing Season**						
Amino acid (A)	1	0.3180	0.3936	0.7164	0.8917	0.2312
Sulphur Doses (D)	4	0.9662	0.7809	0.8933	0.9637	0.3192
A × S	4	0.2548	0.1699	0.7673	0.5888	0.8955
C.V. (5%)		10.82	22.27	5.14	4.27	23.61
Mean		369.37	239.52	36.56	75.9	1717.43
**Faxinal-PR in the 2023 Growing Season**						
Amino acid (A)	1	0.4306	0.3643	0.7779	0.2273	0.2117
Sulphur Doses (D)	4	0.0222 *	0.4763	0.2887	0.5196	0.7102
A × S	4	0.2012	0.4007	0.606	0.2304	0.5708
C.V. (5%)		17.65	20.08	6.24	15.75	14.35
Mean		345	262.07	24.9	65	2194.47

Significant effects are indicated by * at *p* ≤ 0.05. Means followed by different letters within each column differ significantly according to Tukey’s test (*p* ≤ 0.05). Values represent mean ± standard deviation.

**Table 2:** Summary of variance analysis for germination characteristics (%) (G), first germination count (%) (FC), emergence speed index (ESI), shoot length (cm) (SL), root length (cm) (RL), shoot dry mass (g plant^−1^) (SDM), and root dry mass (g plant^−1^) (RDM). Data obtained in the municipalities of Londrina-PR and Sertaneja in the 2022 season, and Londrina and Faxinal in the 2023 season.

Causes of Variation	D.F.	*p*-Value						
		FC (%)	G (%)	ESI	DL (cm)	RL (cm)	SDM (g plant^−1^)	RDM (g plant^−1^)
Londrina-PR in the 2022 Growing Season								
Amino Acid (A)	1	0.762	0.881	0.868	0.499	0.798	0.689	0.252
Sulphur Doses (D)	4	0.183	0.373	0.843	0.947	0.987	0.463	0.335
A × D	4	0.821	0.850	0.812	0.174	0.296	0.788	0.220
C.V.		22.42	9.75	9.06	7.64	20.39	32.21	25.12
Mean		31.71	80.18	63.7	4.89	10.76	0.0888	0.0789
**Sertaneja-PR in the 2022 Growing Season**								
Amino Acid (A)	1	0.072	0.881	0.856	0.777	0.064	0.847	0.251
Sulphur Doses (D)	4	0.004 *	0.373	0.835	0.902	0.079	0.567	0.336
A × D	4	0.066	0.850	0.803	0.376	0.038	0.873	0.453
C.V.		14.58	9.75	9.09	34.00	22.79	25.72	21.27
Mean		26.83	80.18	62.8	11.70	17.85	0.0724	0.0738
**Londrina-PR in the 2023 Growing Season**								
Amino Acid (A)	1	0.684	0.791	0.467	0.819	0.648	0.334	0.347
Sulphur Doses (D)	4	0.974	0.726	0.283	0.890	0.055	0.469	0.392
A × D	4	0.074	0.579	0.787	0.345	0.195	0.621	0.962
C.V.		33.42	9.34	10.28	35.5	34.85	38.27	72.02
Mean		33.26	91.33	67.6	9.74	8.78	0.147	0.0699
**Faxinal-PR in the 2023 Growing Season**								
Amino Acid (A)	1	0.657	0.265	0.486	0.596	0.817	0.934	0.151
Sulphur Doses (D)	4	0.138	0.475	0.181	0.163	0.210	0.659	0.436
A × D	4	0.088	0.481	0.886	0.959	0.188	0.887	0.321
C.V.		15.71	2.82	9.58	17.35	22.95	35.71	20.37
Mean		67.2	92.32	66.97	12.89	15.58	0.0897	0.0878

Significant effects are indicated by * at *p* ≤ 0.05. Means followed by different letters within each column differ significantly according to Tukey’s test (*p* ≤ 0.05). Values represent mean ± standard deviation.

**Table 3:** Summary of variance analysis (*p*-values) for nutrient contents (Nitrogen—N (g kg^−1^), Phosphorus—P (g kg^−1^), Potassium—K (g kg^−1^), Magnesium—Mg (g kg^−1^), Calcium—Ca (g kg^−1^), Sulphur—S (g kg^−1^), Copper—Cu (mg kg^−1^), Iron—Fe (mg kg^−1^), Manganese—Mn (mg kg^−1^) and Zinc—Zn (mg kg^−1^)) in grains under different sulphur doses and amino acid application in the municipalities of Londrina-PR, Sertaneja-PR, and Faxinal-PR during the 2022 and 2023 growing seasons.

Causes of Variation	D.F.	*p*-Value									
		N (g kg^−1^)	P (g kg^−1^)	K (g kg^−1^)	Mg (g kg^−1^)	Ca (g kg^−1^)	S (g kg^−1^)	Cu (mg kg^−1^)	Fe (mg kg^−1^)	Mn (mg kg^−1^)	Zn (mg kg^−1^)
Londrina-PR in the 2022 Growing Season											
Amino Acid (A)	1	0.178	0.187	0.498	0.133	0.021 *	0.710	6.548	0.055	0.290	0.566
Sulphur Doses (D)	4	0.637	0.159	0.769	0.457	0.330	0.507	4.338	0.596	0.508	0.699
A × D	4	0.830	0.749	0.360	0.773	0.173	0.536	9.357	0.228	0.898	0.082
C.V.		84.33	16.86	23.96	7.83	30.93	22.64	23.68	93.88	8.52	5.56
Mean		3.06	2.96	35.40	1.21	1.13	2.21	0.38	89.77	80.70	40.62
**Sertaneja-PR in the 2022 Growing Season**											
Amino Acid (A)	1	0.811	0.718	0.274	0.380	0.882	0.710	0.111	0.118	0.756	0.385
Sulphur Doses (D)	4	0.990	0.185	0.682	0.210	0.001 *	0.507	0.124	0.683	0.401	0.399
A × D	4	0.536	0.202	0.634	0.542	0.202	0.536	0.924	0.455	0.580	0.422
C.V.		50.99	13.18	38.40	10.95	15.69	22.64	44.48	78.24	16.63	41.26
Mean		7.49	3.32	21.94	1.40	1.17	2.22	0.42	91.06	72.94	41.96
**Londrina-PR in the 2023 Growing Season**											
Amino Acid (A)	1	0.107	0.062	0.238	0.152	0.502	0.642	0.210	0.142	0.208	0.536
Sulphur Doses (D)	4	0.144	0.806	0.572	0.992	0.963	0.717	0.671	0.538	0.930	0.698
A × D	4	0.585	0.357	0.679	0.727	0.641	0.342	0.422	0.412	0.584	0.791
C.V.		67.75	24.28	26.94	13.81	19.54	24.03	37.11	83.75	26.94	39.59
Mean		21.40	3.64	30.82	1.35	1.25	2.00	0.45	161.19	58.24	39.19
**Faxinal-PR in the 2023 Growing Season**											
Amino Acid (A)	1	0.479	0.846	0.480	0.415	0.441	0.135	0.447	0.251	0.491	0.576
Sulphur Doses (D)	4	1.000	0.469	0.563	0.397	0.537	0.910	0.404	0.682	0.168	0.176
A × D	4	0.976	0.491	0.114	0.898	0.235	0.255	0.351	0.514	0.670	0.609
C.V.		47.29	23.75	44.73	7.28	22.61	11.22	41.16	44.39	63.59	61.36
Mean		12.57	3.96	26.18	1.40	1.46	2.09	0.43	51.52	23.43	30.10

Significant effects are indicated by * at *p* ≤ 0.05. Means followed by different letters within each column differ significantly according to Tukey’s test (*p* ≤ 0.05). Values represent mean ± standard deviation.

**Table 4:** Chemical characterization of the soils used in the experiment in the municipalities of Londrina-PR, Sertaneja-PR, and Faxinal-PR. 2022/2023. Interpretation according to the Manual of fertilization and liming of the state of Paraná [63].

Soil Chemical Characteristics											
Local	pH	MOS	Ca^2+^	Mg^2+^	Al^3+^	P	K^+^	CEC	V	S	B
	CaCl_2_	g dm^−3^	cmolc dm^−3^			mg dm^−3^	cmolc dm^−3^		%	mg dm^−3^	
Londrina-PR 2022	4.2	9.3	4.25	1.14	0	13.6	0.56	10.9	64.1	13	0.49
Level	B	B	A	A	B	MA	MA	M	A	MA	M
Sertaneja-PR 2022	5.08	22.94	5.97	1.8	0	37.61	0.4	14.5	56.3	31.1	0.77
Level	A	M	A	A	B	MA	M	M	A	MA	MA
Londrina-PR 2023	4.8	10.3	6.33	1.5	0	11.6	0.53	12.3	67.9	13	0.49
Level	M	M	MA	A	B	A	MA	M	A	MA	M
Faxinal-PR 2023	4.82	49.49	3.51	1.46	0.47	18.87	0.25	5.72	39.8	7.72	0.83
Level	M	A	A	A	B	MA	M	B	B	MA	MA

Source: adapted from Pauletti, [63]. (B: low, A: high, M: medium, MA: very high).

## Data Availability

The original contributions presented in this study are included in the article. Further inquiries can be directed to the corresponding author.

## Supplementary Materials

The following supporting information can be downloaded at: https://www.mdpi.com/article/10.3390/plants15010066/s1. Table S1. Amino acid profile and free amino acid profile present in bovine collagen hydrolysate obtained from the chemical hydrolysis of animal protein. Values are expressed as percentages for amino acids and mg/g for total collagen. Source: adapted from Eurofins, 2023.
