# Supplementary material for: Foliar Nanoparticulate Sulphur and Amino Acids Modulate Wheat Yield Components and Seed Quality Across Contrasting Environments
Source: Plants (Basel). 2025 Dec 25;15(1):66. doi: 10.3390/plants15010066 (PMC12787486; doi:10.3390/plants15010066)
Supplement: Supplementary file 1 [file plants-15-00066-s001.zip › plants-3988252-supplementary.pdf]

plants

Supplementary Materials: The following supporting information can be downloaded at: https://www.mdpi.com/article/doi/s1.

Table S1. Amino acid profile and free amino acid profile present in bovine collagen hydrolysate obtained from the chemical hydrolysis of animal protein. Values are expressed as percentages for amino acids and mg/g for total collagen. Source: adapted from Eurofins, 2023.

**Amino Acid Profile**			**Free Amino Acid Profile**		
**Parameter**	**Results**	**Unit**	**Parameter**	**Results**	**Unit**
Ácido Aspártico	3.07	%	Ácido Aspártico	0,05	%
Ácido Glutâmico	5.62	%	Ácido Glutâmico	0.09	%
Alanina	5.73	%	Alanina	0.32	%
Arginina	2.02	%	Arginina	<0.01	%
Fenilalanina	1.12	%	Fenilalanina	0.03	%
Glicina	13.49	%	Glicina	1	%
Histidina	0.41	%	Histidina	<0.01	%
Isoleucina	0.75	%	Isoleucina	0.88	%
Leucina	1.82	%	Leucina	<0.01	%
Lisina	1.85	%	Lisina	0.03	%
Prolina	8.26	%	Prolina	0.22	%
Serina	0.36	%	Serina	0.24	%
Tirosina	<0.04	%	Tirosina	<0.01	%
Treonina	0.23	%	Treonina	0.01	%
Valina	1.49	%	Valina	<0.01	%
Cistina + Cisteína	0.03	%	Cisteína	0.02	%
Metionina (total)	0.54	%	Metionina	0.07	%
Colágeno	360	mg/g	Colágeno	360	mg/g
